# Rethinking abortion law reform in Malawi: a multi-method study

**DOI:** 10.1080/26410397.2026.2702233

**Published:** 2026-07-27

**Authors:** Carmen Hall, Judith Daire, Delia Hendrie, Genesis Chorwe-Sungani

**Affiliations:** a Postgraduate student, School of Population Health, Curtin University, Perth, Australia.; b Senior Lecturer, School of Population Health, Curtin University, Perth, Australia; c Associate Professor, School of Population Health, Curtin University, Perth, Australia; d Associate Professor, School of Nursing, Kamuzu University of Health Sciences, Blantyre, Malawi

**Keywords:** abortion law, abortion policy, Malawi, unsafe abortion, maternal mortality, reproductive health and rights, low-income country, Africa

## Abstract

Despite decades of sustained advocacy, Malawi retains one of the world’s most restrictive criminal abortion laws. A draft Termination of Pregnancy Bill, developed in 2015 to propose semi-liberal reform to Malawi’s abortion law, has yet to be enacted. In contrast, similar or less intensive advocacy efforts have resulted in liberalisation of abortion laws in other low- and middle-income countries (LMICs), including Zambia, South Africa, and Ethiopia. This qualitative case study examines the policy processes adopted by advocates and identifies potential pathways for abortion law reform in Malawi. Employing a multi-method approach, we conducted document analysis using the READ approach and semi-structured interviews with 14 key informants actively or formerly involved in Malawi’s abortion law reform process. Data was analysed thematically using Bacchi’s (2009) “What’s the problem represented to be?” (WPR) theoretical framework. The findings revealed evidence-informed strategies aimed at strengthening future advocacy efforts: coordinated advocacy integrating established and emerging organisations; ongoing demographic and lived-experience evidence to counter stereotypes and expose harms; careful reframing of language (e.g. replacing “Termination of Pregnancy Bill” with motherhood-aligned titles); intensive community engagement via trusted local champions; strategic timing mindful of political cycles and U.S. funding pressures (including the reinstated Global Gag Rule); further litigation to clarify and expand existing legal scope; and adequate funding to counter well-resourced opposition and sustain rural outreach. Drawing comparative insights from surrounding Sub-Saharan African countries, we discuss drivers of reform and propose targeted strategies to advance abortion law liberalisation in Malawi and reduce mortality related to unsafe abortion. *DOI: 10.1080/26410397.2026.2702233*

## Plain language summary

Malawi's very strict abortion law is one of the most restrictive in the world. For many years, people in Malawi have campaigned hard to change the law. The draft Termination of Pregnancy Bill was created in 2015 to make the law more liberal, but Parliament has still not passed it. Other countries like Zambia, South Africa and Ethiopia, have changed their laws with similar campaigns. This study looked closely at how advocates in Malawi have been working to change the law and what could help them succeed. We reviewed documents and spoke with 14 people who have been involved in the campaign. We used a framework called the “What’s the Problem Represented to be?” approach to analyse what they said and found. The study identified strategies which could prove valuable in advocating for change. 1) Older and newer advocacy groups could coordinate better. 2) Maintain up-to-date evidence about the effects of the current law, including real-life stories of the harm it causes. 3) The proposed bill title could use more socially acceptable wording. 4) Advocates should work closely with trusted local leaders. 5) Timing the right moment to push the bill in relation to elections and U.S funding restrictions is important. 6) Court cases would clarify what the current law already allows. 7) Campaigns need to secure enough money to match the resources of groups opposed to change. Our study looked at what has worked in nearby African countries. We suggest steps that could help progress legal change in Malawi and reduce deaths from unsafe abortion.

## Résumé

En dépit de décennies de plaidoyer soutenu, le Malawi conserve l’une des législations les plus restrictives au monde en matière d’avortement. Un projet de loi sur l’interruption de grossesse, élaboré en 2015 pour proposer une réforme semi-libérale de la loi malawienne sur l’avortement, n’a toujours pas été promulgué. À l’inverse, des activités de plaidoyer similaires ou moins intensives ont abouti à une libéralisation des lois sur l’avortement dans d’autres pays à revenu faible ou intermédiaire, notamment en Zambie, en Afrique du Sud et en Éthiopie. Cette étude de cas qualitative examine les processus politiques adoptés par les acteurs du plaidoyer et identifie des pistes potentielles pour une réforme de la loi sur l’avortement au Malawi. À l’aide d’une approche à méthodes multiples, nous avons mené une analyse documentaire selon la méthode READ et des entretiens semi-structurés avec 14 informateurs clés participant ou ayant participé activement au processus de réforme législative au Malawi. Les données ont été analysées thématiquement en utilisant le cadre théorique de Bacchi (2009), à savoir « Quelle est la représentation du problème? ». Les résultats ont révélé des stratégies fondées sur les données probantes visant à renforcer les futures activités de plaidoyer: un plaidoyer coordonné intégrant les organisations établies et émergentes; des données démographiques et des témoignages continus pour lutter contre les stéréotypes et exposer les préjudices; une reformulation soigneuse des termes employés (par exemple remplacer « projet de loi sur l’interruption de grossesse » par des expressions centrées sur la maternité); une participation intensive de la communauté par le biais de personnalités de confiance; un calendrier stratégique tenant compte des cycles politiques et des pressions de financement des États-Unis (notamment le rétablissement de la règle du bâillon mondial); de nouvelles actions en justice pour clarifier et élargir le champ juridique existant; et un financement suffisant pour contrer une opposition bien dotée en ressources et soutenir les actions en milieu rural. Nous appuyant sur des données comparatives de pays voisins d’Afrique subsaharienne, nous analysons les facteurs favorisant la réforme et proposons des stratégies ciblées pour faire avancer la libéralisation de la loi sur l’avortement au Malawi et réduire la mortalité maternelle liée aux avortements non médicalisés.

## Resumen

A pesar de décadas de incidencia política continua, Malaui mantiene una de las leyes penales sobre aborto más restrictivas del mundo. Aún no se ha promulgado el anteproyecto de ley sobre la Interrupción del Embarazo, redactado en 2015 para proponer una reforma semi-liberal de la ley de Malaui referente al aborto. En cambio, similares o menos intensivos esfuerzos de incidencia política han propiciado la liberalización de las leyes sobre aborto en otros países de bajos y medianos ingresos (PBMI), entre ellos Zambia, Sudáfrica y Etiopía. Este estudio de caso cualitativo examina los procesos de políticas adoptados por promotores e identifica posibles vías para la reforma de la ley sobre aborto en Malaui. Aplicamos un enfoque multimétodo para realizar un análisis de documentos utilizando el enfoque READ y entrevistas semiestructuradas con 14 informantes clave involucrados activa o anteriormente en el proceso de reforma de la ley de Malaui referente al aborto. Se analizaron los datos temáticamente utilizando el marco teórico de Bacchi (2009) “¿Cuál parece ser el problema?” (“*What’s the problem represented to be?* o WPR). Los hallazgos revelaron estrategias informadas por evidencias destinadas a reforzar los futuros esfuerzos de incidencia política: incidencia política coordinada para integrar organizaciones establecidas y emergentes; evidencia continua demográfica y de vivencias para contrarrestar los estereotipos y exponer los daños; una reformulación cuidadosa de la terminología (p. ej. reemplazar el título “Proyecto de ley sobre la Interrupción del Embarazo” con títulos alineados con la maternidad); participación comunitaria intensiva vía defensores locales de confianza; sincronización estratégica en armonía con los ciclos políticos y las presiones de financiación de Estados Unidos (entre ellas la Ley Mordaza restablecida); más litigio para aclarar y ampliar el alcance legislativo actual; y financiación adecuada para contrarrestar una oposición bien financiada y sostener el alcance comunitario rural. Basándonos en perspectivas comparativas de países circundantes de África subsahariana, analizamos los impulsores de la reforma y proponemos estrategias focalizadas para promover la liberalización de la ley sobre aborto en Malaui y reducir la mortalidad relacionada con el aborto inseguro.

## Introduction

Much of the world has witnessed a significant liberalisation of abortion laws over the past century. Since the 1994 International Conference on Population and Development (ICPD), several low- and middle-income countries (LMICs), including in the region of Sub-Saharan Africa, have expanded the legal grounds for abortion.^1^ According to a 2020 Guttmacher Institute report, approximately 44% of Sub-Saharan African countries (21 out of 48) expanded the grounds on which abortion is legal between 2000 and 2019.^2^ This includes countries such as Ethiopia (2005), Rwanda (2012) and Mozambique (2014). Despite decades of sustained advocacy efforts, Malawi retains one of the world’s most restrictive criminal abortion laws.^3^ A relic of colonisation, abortion law in Malawi is based on the United Kingdom’s Offences Against the Person Act 1861. Enacted in 1929, Section 243 of Malawi’s Penal Code states that abortion is only permitted if it is necessary to preserve the life of the pregnant woman. Medical abortion is excluded from the current (1929) law and therefore illegal under all circumstances.^3,4^ Section 150 of Malawi’s Penal Code states that attempting to procure an abortion is a felony and attracts criminal penalties between seven and fourteen years imprisonment.^5^ Despite legal restrictions and criminalisation, women and girls continue to seek abortions, often through unsafe and clandestine means.^3^ It should be noted that abortion exists along a continuum of risk; thus, not all illegal abortions are unsafe, and safer methods such as self-managed medical abortion in accordance with WHO guidelines may still occur despite legal restrictions.^6^ However, the vast majority of abortions in Malawi occur under unsafe conditions, making unsafe abortion one of the leading direct causes of maternal mortality.^7^

Malawi continues to have one of the highest maternal mortality ratios in the world, with an estimated 225 deaths per every 100,000 live births in 2023.^8^ This rate is substantially higher than the global average of 197 deaths per 100,000 live births.^8^ Local empirical evidence has consistently shown that the country’s highly restrictive abortion law is a key contributing factor to elevated rates of unsafe abortion and poor maternal health outcomes.^9,10^ In 2015, an estimated 141,044 induced abortions occurred in Malawi, translating to a national rate of 38 abortions per 1,000 women aged 15–49.^7^ Many of these procedures involved dangerous and invasive methods, such as ingesting poisonous substances (including detergent) or inserting crude objects like cassava stems or bicycle spokes.^11^ As a result, an estimated 51,693 women sought post-abortion care for complications including sepsis, retained products of conception, uterine perforation and permanent infertility in 2015.^10,12^ A 2009 study further found that over 80% of those seeking post-abortion care were married, many of whom delayed seeking treatment due to fear of legal repercussions and stigma.^12^

These poor outcomes are exacerbated by broader reproductive health challenges. The 2017 Demographic and Health Survey reported a total fertility rate of 4.2 for women aged 15–49, with 41% of births unplanned and a 19% unmet need for family planning.^13,14^ Although modern contraceptive prevalence has risen to approximately 66% among married women in 2024, uptake remains considerably lower (37–42%) among unmarried and sexually active young women, as shown.^15^ Low levels of health literacy among women, particularly in rural areas where female literacy hovers around 62–68%, further compound these challenges by limiting women’s ability to access and effectively use modern contraceptive methods.^15^

There has been sustained and growing pressure from international and regional human rights and public health bodies for Malawi to address its high rates of unsafe abortion. While the 1994 ICPD Programme of Action emphasised the need for post-abortion care to mitigate the public health consequences of unsafe abortion, more recent pressure has come from specific global and regional mechanisms.^16^ As a signatory to the Maputo Protocol and the Convention on the Elimination of All Forms of Discrimination against Women (CEDAW), Malawi has faced repeated calls from the CEDAW Committee and the African Commission on Human and Peoples’ Rights to expand access to safe abortion care and align its laws with its regional and international commitments.^17–19^ These bodies have consistently urged the Malawian government to reform its highly restrictive colonial-era law, primarily on public health and human rights grounds.

In 2013, the Malawian government established a Special Law Commission (SLC) to review the country’s abortion law. The SLC included representatives from the Ministries of Health and Justice, local government, various religious groups, the Malawi College of Medicine and the Malawi Law Society. Its formation was primarily driven by sustained advocacy from Ipas Malawi and the Coalition on Prevention of Unsafe Abortion (COPUA), supported by compelling local evidence on the burden of unsafe abortion and maternal mortality.^4,5^ Drawing on consultations conducted across 12 of the 28 districts and national evidence, the SLC’s findings informed the drafting of the Termination of Pregnancy Bill (ToP) in 2015.^20,21^ Throughout the consultation process, religious stakeholders – particularly members of the Catholic Church – articulated strong moral opposition to abortion.^4^ It was also evident that most Malawians considered sexual and reproductive health taboo, especially the topic of abortion.^10^ Members of the SLC therefore believed that the largely conservative society of Malawi would not support radical abortion law reform, such as full decriminalisation or allowing for abortion on request. This led the Commission to favour a semi-liberal law – one that would expand legal access to abortion under specific grounds such as risk to the woman’s physical or mental health, rape, incest, and severe foetal impairment, while still prohibiting abortion on request or for socio-economic reasons – as more socially acceptable and therefore more likely to be passed through Parliament.^3^

Initially, the ToP Bill received notable cross-party support as well as endorsements from a range of stakeholders, including civil society networks, senior officials in the Ministry of Health and some religious leaders.^22^ Several prominent religious bodies, such as the Malawi Council of Churches, the Episcopal Conference of Malawi (Roman Catholic Church) and the Muslim Association of Malawi, who were all integral partners in the commissioning process, provided written endorsement of the proposed bill.^3^ However, many of these same institutions later withdrew their support and publicly voiced strong opposition to any liberalisation of the law.^3^ Despite this shifting position among some religious leaders, local advocates – particularly the COPUA and allied women’s rights and health organisations – continued to engage in intense and sustained advocacy. This included targeted meetings with parliamentarians and traditional leaders, values clarification workshops with religious groups, strategic use of local public health evidence, and broad media engagement to raise awareness.^3^ An initial attempt to introduce the bill in Parliament in 2016 was rejected amid public protests, while a second attempt in March 2021, led by Hon. Dr Matthews Ngwale, (then Chairperson of the Parliamentary Committee on Health), was blocked by an intervening motion that was widely viewed as a deliberate tactic by opposing groups.^3^ Currently, there is a widespread lack of open public support for comprehensive abortion law reform, largely shaped by deeply held religious and conservative cultural values. This has created considerable uncertainty and reluctance among many parliamentarians to advance the bill.^21^ As of July 2026, the ToP Bill is still pending in Parliament, with no further progress since the unsuccessful attempt to table it in 2021.

In response to persistent calls from local advocates and the need to address inconsistencies in service provision, the Ministry of Health introduced the Standards and Guidelines on Post-Abortion Care in 2020. These guidelines were driven by efforts to provide clearer direction to healthcare providers on the limited legal exception (to save the woman’s life) and to improve the quality of post-abortion care amid high rates of complications from unsafe abortions.^5^ However, while they offer some operational clarity, the guidelines have not altered the underlying highly restrictive legal framework. Local advocates, including the COPUA, have continued to work alongside these initiatives, combining public health evidence with strategic engagement of parliamentarians to push for broader reform. Despite this combined domestic and international pressure, successive governments have been reluctant to advance legislative change, largely due to strong religious and cultural opposition within the country.

Previous studies have shown that similar advocacy efforts for abortion law reform have been witnessed in other LMICs, including the Sub-Saharan Africa (SSA) countries of Ethiopia, South Africa and Zambia.^23–26^ In Malawi, as in these countries, the process has been shaped by both domestic and transnational advocacy involving actors supporting and opposing reform.^21^ Local NGOs and women’s rights organisations have played a central and sustained role in pushing the reform agenda. These domestic efforts have been complemented by technical and financial support from international donor-funded organisations such as Ipas and Marie Stopes International (MSI).^22^ Yet, despite this combined local and international advocacy, Malawi continues to retain one of the most restrictive abortion laws in the region. The Malawian case therefore offers a valuable opportunity to retrospectively examine the policy process and identify strategic areas for future focus on the path towards reform. The aim of this study was to identify potential pathways for successful abortion law reform in Malawi, drawing on comparative insights from other Sub-Saharan African countries. Using a multi-method approach, which included both document analysis and investigative interviews, we reviewed the policy processes adopted by advocates in favour of reform as well as the influence of those resistant to change. We also explored practical strategies for the advancement of more progressive abortion laws in Malawi, informed by the experiences of countries in the region such as Ethiopia, Rwanda, South Africa, Mozambique and Zambia.

### Theoretical framework

Policy analysis is essential for multi-disciplinary inquiry, helping to explain reasons for the success or failure of policies and to provide a roadmap for future action. This study utilised Carol Bacchi’s What’s the Problem Represented to be? (WPR) theoretical framework.^27^ This framework approaches policy analysis through an unconventional lens, whereby policies are not viewed as simply responding to pre-existing problems or issues; instead, they actively create or contribute to the problem itself.^28^ The WPR framework includes a set of six overarching questions (Table 1), which guided the development of the study, including interview question design, data extraction and analysis. Using the six questions of the WPR framework allowed for deeper inspection into what assumptions the problem rests on, what is ignored, and the real-world effects on people’s lives.^27^ The WPR framework has been designed as a versatile tool that can be applied across many policy fields, including health policy.^28^ The framework can also be used to analyse interviews; however, this approach is less commonly used relative to document analysis. Applications of the WPR framework to abortion policy include analyses of restrictive abortion laws in LMICs^29^ and post-abortion contraception in the UK.^30^

**Table 1.** Reader guide

Study objectives	WPR questions		Paragraph themes	Sub-themes
Review the policy process adopted by advocates in favour of reform	1	What is the “problem” represented to be?	Public health issue	** **
	2	What presuppositions or assumptions underlie this representation of the “problem”?	Deep-rooted morality and religious values	
	3	How has this representation of the “problem” come about?	International frameworks on sexual and reproductive health and rights	
	4	What is left unproblematic in this problem representation? Where are the silences?	Other arguments for reform	Women’s rights
				Gender discrimination
				Parliamentary dynamics
	5	What effects are produced by this representation of the “problem”?	Consequences of the public health framing	Narrowing the debate to public health
				Marginalisation of rights and gender equality arguments
				Shaping advocacy strategies
				Influencing political acceptability
				Obscuring structural and social dimensions
Explore strategies for the advancement of more progressive or liberal abortion laws in Malawi	6	How and where is this representation of the “problem” produced, disseminated, and defended? How could it be questioned, disrupted, and replaced?	Potential pathways for successful abortion law reform in Malawi	Coordinated approach
				Further evidence
				Language
				Stakeholder Engagement
				Timing
				Litigation
				Funding
				Path to comprehensive law reform

## Methodology

With a view towards constructing a holistic account of the abortion law reform process in Malawi, we employed a multi-method case study design using qualitative evidence. First, we collected secondary data through document analysis of peer-reviewed journal articles and grey literature (reports and media articles). Second, we collected primary data through semi-structured interviews with key informants.

### Document analysis

We applied the READ (Ready, Extract, Analyse, Distil) approach to the document analysis, which provides a step-by-step guide for qualitative policy research.^31^ The steps consist of: (1) ready materials, (2) extract data, (3) analyse data and (4) distil findings.^31^ In step one, the primary researcher (CH) used purposive sampling to identify key policy and related contextual documents. Documents were identified by (i) searching for peer-reviewed and grey literature, (ii) reference tracking, and (iii) suggestions by interview participants. Data collection for documents occurred between October and April 2024 and was limited to those published in English. Retrieved documents primarily focused on the topics of unsafe abortion and abortion law reform in Malawi. To monitor major events related to abortion law in Malawi, we also scanned digital media via Google News. This was limited to news articles published in English, using a date range from 2013 (commencement year of the SLC) to 2024. We excluded documents which included repetitive information (i.e. content that duplicated what had already been obtained from other sources without providing new insights), lacked specific information on unsafe abortion or had a non-Malawi focus. Document acquisition was enhanced by asking for documents during key informant interviews.

Following on with the four-step READ approach, step two involved extracting data on reference information, document titles, type of document, key points and website links into a customised Microsoft Excel spreadsheet. Initially, 30 documents were identified and 21 were selected for full analysis. Of the 21 included documents, 8 were peer-reviewed. The documents included were published over 13 years (2011–2024) and considered advocacy from differing perspectives, parliamentary developments, or academic research on the topic of abortion in Malawi. The peer-reviewed documents discussed abortion advocacy, incidence, transnational influence, social consequences, human rights or abortion law. We identified 20 documents purely focused on Malawi, and one multi-region document (including Malawi). The selected documents were then imported into Nvivo, qualitative data analysis software which supports both inductive and deductive coding. In step three, the researchers met to agree on initial thematic codes using the WPR framework as a guide. These codes were entered in Nvivo and continuously refined throughout the analysis stage. The primary researcher (CH) read and re-read all included documents; data was first coded deductively using the WPR framework, followed by inductive coding to identify additional categories. In step four, we stopped the document review when no additional new information could be obtained from further analysis.

### Key informant interviews

We identified an initial list of potential interviewees throughout the document analysis process. An Excel document was used to record names, associations and contact information where available. The researchers sent an expression of interest letter via email to: (i) authors of included policy documents and journal articles, (ii) individuals advocating for abortion law reform and (iii) additional individuals recommended by interview participants. Interested individuals contacted the researchers, who then sent participant information statements, answered questions, and arranged interview times with those willing to proceed. Altogether, 31 key informants were initially contacted, 15 did not respond and two declined.

Prior to being interviewed, each participant received a written information statement which explained the purpose of the study and their involvement. They were then asked to read and sign a written consent form. In addition, a second verbal consent was requested prior to audio recording the interviews. Participants were assured of confidentiality and reminded that their involvement in the study was voluntary and that they could stop the interview or withdraw their consent to have their data used in the research at any time. As the researchers and interviewees were based in remote settings, all interviews were conducted virtually over the Microsoft Teams call platform. All interviews were conducted individually and in English (official language of Malawi). We initially used purposive sampling followed by snowball sampling to expand the number of informants, making efforts to secure participants from a broad range of sectors and to include different points of view. The sample size for the qualitative interviews was guided by the concept of information power.^32^ This approach suggests that the richer and more relevant the information held by participants in relation to the study’s specific aims and research question, the fewer participants are required.

Interviews were conducted between February 2024 and April 2024 using a semi-structured interview guide with open-ended questions based on the WPR framework. Although there were some common questions asked of most interviewees, the researchers did not employ a uniform survey instrument, since each interviewee had unique knowledge, given their professional backgrounds. Instead, we designed the interview questions to be exploratory, providing for focused discussion whilst also allowing flexibility for emerging conversations based on individual knowledge and experience.

In total, 14 key informants were interviewed, lasting between 16 and 47 minutes (mean 30 minutes). We achieved a maximum variation sample representing diversity of gender (7 female, 7 male), employment sectors (public, private, non-profit sector), and direct experience in the abortion law reform process in Malawi. Professional backgrounds included academia, law, media, reproductive health professionals, Ministers of Parliament, human/women’s rights NGOs, INGOs, Ministry of Health and Reproductive Health Directorate. At the time of conducting the interviews, 4 informants had already retired. However, many had years of experience in the field, with 3 out of 14 interview participants having been directly involved in the development of the Report of the Law Commission on the Review of the Law on Abortion in Malawi. Recorded interviews were transcribed verbatim. One researcher (CH) went through the transcripts, comparing them against the audio recording to confirm the correctness of the transcriptions. The transcripts were provided to informants following the interview, providing the opportunity for review. To protect the identity of the informants, the transcriptions were anonymised. Finalised transcripts were imported into NVivo and coded deductively.

### Reflexivity and ethics

This work builds on previous research on the processes involved in changing abortion laws in the broader context of LMICs.^23^ CH is an Australian PhD student at Curtin University in Western Australia. As the primary researcher, she searched for documents, interviewed key informants, extracted data, analysed the data and drafted the manuscript with critical peer review from three co-supervisors who are also co-authors (JD, GCS, and DH). JD is originally from Malawi, currently working in Australia since 2015, and is a senior lecturer in health policy and a secondary supervisor. DH, from Australia, is an associate professor in health economics and the primary supervisor. GCS is currently living and working in Blantyre, Malawi. He is an executive dean for the school of nursing at the Kamuzu University of Health Sciences and associate professor in mental health, providing supervision for the study.

Ethical approval for collecting primary interview data was obtained from the Curtin University Human Research Ethics Committee on the 27 September 2023 (HRE2023-0549) and the University of Malawi – College of Medicine Research and Ethics Committee on the 21 November 2023 (P.08/23-0220). Ethical approval was only required for the interview component of the research as documents were searched using publicly available databases. The 21-item checklist from the Standards for Reporting Qualitative Research (SRQR) was used to inform the qualitative approach of the research and to report on the study findings.^33^

## Findings

This analysis, guided by the WPR framework and its six guiding questions, examines the policy processes and strategies employed by advocates seeking abortion law reform in Malawi. The findings begin by identifying the dominant representation of the “problem”, whereby abortion law reform has been predominantly represented as a public health issue within policy discourse in Malawi. We then explore the underlying presuppositions rooted in Malawi’s entrenched religious and cultural values, the influence of international sexual and reproductive health and rights frameworks, and the surrounding alternative arguments centred on women’s rights, gender discrimination and parliamentary dynamics. Next, we examine the effects produced by this dominant public health representation, including the narrowing of the policy debate, the marginalisation of rights-based arguments, the shaping of advocacy strategies, its influence on political acceptability, and the obscuring of deeper structural and social drivers such as gender inequality, poverty and pervasive stigma. Finally, we outline potential pathways for advancing reform, including the need for a coordinated advocacy approach, further demographic evidence, the use of language, effective stakeholder engagement, appropriate timing to reintroduce the ToP Bill, strategic litigation, funding dynamics and the long-term trajectory towards comprehensive abortion law liberalisation.

### What is the “problem” represented to be?

#### Public health issue

In this analysis, the “problem” is understood as the representation of abortion law reform primarily as a public health issue. Advocates in Malawi have strategically framed abortion law reform as an urgent public health intervention required to address the country’s persistently high maternal mortality ratio. This representation draws on robust local evidence demonstrating the scale and severe consequences of unsafe abortion, including high rates of complications, permanent injury and preventable deaths.^7,12^ Public health arguments have proven more culturally and politically acceptable in Malawi’s conservative context than those that directly challenge prevailing social, moral and religious norms.^3,21^ As a result, locally generated evidence highlighting the preventable harms of unsafe abortion has become a central tool in advocacy campaigns. By presenting the issue primarily as a technical and solvable health crisis rather than a rights-based or moral one, advocates have sought to build broader support for the proposed ToP Bill. However, despite this pragmatic framing, religious institutions and the anti-abortion movement continue to mount strong and organised resistance, often prioritising moral and foetal rights arguments over public health considerations.

### What presuppositions or assumptions underlie this representation of the “problem”?

#### Deep-rooted morality and religious values

Given Malawi’s entrenched religious and cultural values, utilising a public health argument has been the favoured approach, largely because it is seemingly the least antagonising argument. The Special Law Commission (SLC) deliberately involved diverse representatives to reflect fair representation of Malawian society.^4^ Members from religious associations and councils initially endorsed the SLC recommendations and proposed ToP Bill.^3^ Yet, following public release of the proposed bill, those same institutions denounced any change to the law and have since maintained strong resistance against abortion law reform.^34^

The strides towards reform, including the SLC and drafting of the ToP Bill, occurred when the two preceding presidents, Joyce Banda and Peter Mutharika, were in power, both of whom were committed to improving women’s status and reproductive health.^11^ However, both attempts to table the ToP Bill in 2016 and 2021 were blocked due to strong opposition from religious leaders and institutions within the political arena. Former President Lazarus Chakwera, for example, previously led the Malawi Assemblies of God evangelical church. One informant suggested that the difference between Malawi and its neighbouring countries, such as Zambia and Mozambique, may be the strength of religious voices in Parliament alongside the fear of losing their parliamentary positions. While advocates acknowledged the considerable influence of religiosity, they questioned the consistency of parliamentarians and lawmakers in their support for reform:

“*They’re not acting as though it’s a terrible crime … Basically, they’re pretending that they’re religiously and morally objected to it, but the actual situation on the ground is quite the contrary … I also think that it's a bit rich to say it’s based on their religious and moral objections when you know there are 140,000 abortions per year, and nobody gets prosecuted, they're all pretending it [abortion]’s a terrible crime.*” (KI7 – legal professional)

### How has this representation of the “problem” come about?

#### International frameworks on sexual and reproductive health and rights

Malawi has faced growing pressure from key international human rights and health bodies, including the CEDAW Committee, the World Health Organization (WHO) and the African Commission on Human and Peoples’ Rights, to address the public health consequences of unsafe abortion and align its laws with global and regional standards for safe abortion care. As a signatory to the Convention on the Elimination of All Forms of Discrimination Against Women (CEDAW) and the Maputo Protocol, Malawi has been repeatedly urged by the CEDAW Committee to review and amend its penal code by removing punitive provisions, and to ensure women have access to safe abortion services – at minimum in cases where the pregnancy endangers the woman’s life or health, results from rape or incest or involves foetal impairment – along with quality post-abortion care. Article 14(2)(c) of the Maputo Protocol further obliges states to provide appropriate medical measures for abortion in cases of sexual assault, rape, incest or risks to mental and physical health.^5,9^ However, Malawi’s current law remains non-compliant with these obligations. The SLC explicitly highlighted these inconsistencies with both the national Constitution and international human rights treaties.^4^ A major hurdle remains in the domestic processes required to translate Malawi’s international commitments into enforceable national law. Although Malawi has ratified key instruments such as CEDAW and the Maputo Protocol, these treaties must still undergo parliamentary tabling and domestication to become fully operational within the country’s legal system. Following ratification of the Maputo Protocol, the government took some steps towards domestication; however, only partial incorporation has occurred, particularly with respect to the abortion-related provisions.^35^ This has resulted in a continued gap between Malawi’s international obligations and its highly restrictive penal code, despite the SLC’s efforts to highlight these inconsistencies with both the national Constitution and regional human rights standards.^4^

### What is left unproblematic in this problem representation? Where are the silences?

#### Other arguments for reform

##### Women’s rights

While the public health burden has been a primary focus, advocates have also wielded arguments based on women’s and reproductive rights. In addition to the recognition of women’s rights in Malawi’s own constitution, the 2013 Gender Equality Act also affirms access to sexual and reproductive health services, including choice over the number and timing of children.^36^ Rights are often dismissed as Western or incompatible with Malawian values and are often countered by claims of foetal rights: “*when we have tried to use a rights argument, the question has always been, why honour the rights of the women? Why not the rights of the unborn baby as well?”* (KI2 – health professional).^4^ Foetal rights have never been determined or recognised by regional or international human rights treaties.^4^ Opponents also argue that access to safe abortion care would promote promiscuity and would be used as a form of family planning, yet evidence demonstrates that liberalisation reduces abortion rates.^4,37^ Rights arguments have reframed the conversation on abortion in contexts where moralistic or religious arguments exist, such as throughout the region of Latin America, despite dominant views centred on protecting life from conception.^23,38,39^

##### Gender discrimination

Current law discriminates by denying women a lifesaving service that only they require.^4^ The Southern African Litigation Centre deems state failure to ensure access to legal abortion constitutes gender discrimination.^40^ As one advocate described: “*it’s a very important issue for women’s equality rights and you know, if men were being killed at this rate by a medical procedure, I think there would be a very different outcome*” (KI7 – legal professional). However, many feared such arguments would only antagonise religious opponents and would discourage open dialogue between parliamentarians.

##### Parliamentary dynamics

Historically, the governments supported reform, beginning with the Ministry of Health requesting technical support from Ipas and the World Health Organization (WHO) to conduct a strategic assessment on the problem of unsafe abortion in Malawi.^3^ This was followed by a comprehensive nation-wide study on unsafe abortion, providing significant data that helped to initiate civil society advocacy in 2010.^3^ However, more recently lawmakers have avoided open discussions with constituents:

“*When you ask them, they say no, I cannot say that in my constituency, you advocates are the ones who need to come to our constituencies, talk with the Chiefs, talk with every level of the community. So, it's like on one [hand] they understand that unsafe abortion complications are killing our women. But on the other hand, they are not ready to be on the forefront, to change the law.*” (KI5 – Ministry of Health)

Hon Dr Matthews Ngwale, who courageously attempted to present the bill, expressed uncertainty about the process, “we are prescribing to the people, in other words, we are telling people what they should have, people are not telling us what they want, that’s where the disconnect is”.^41^ This reflects a cloud of confusion and lack of ownership surrounding the ToP Bill. Ultimately, passage of the bill rests with Malawian MPs. Although private meetings often elicit open understanding and support for reform, that backing quickly fades upon return to Parliament. Promising champions face intimidation from party leaders to follow party lines against change, while religious authorities have threatened to withhold votes from supportive parliamentarians.

### What effects are produced by this representation of the “problem”?

#### Consequences of the public health representation

By predominantly framing unsafe abortion as a public health crisis, this representation has shaped both the scope of policy responses and the dimensions of the issue that are foregrounded or backgrounded. While this framing has produced some strategic advantages, it has also generated several limiting effects.

##### Narrowing the debate to public health

Framing abortion law reform primarily as a public health issue centres the discussion on maternal morbidity and mortality, positioning unsafe abortion as a technical health problem requiring intervention.^23^ While this approach has enabled the use of locally resonant evidence and facilitated engagement with policymakers, it has also narrowed the scope of debate, limiting attention to measurable health outcomes rather than broader social, legal and ethical considerations.

##### Marginalisation of rights and gender equality arguments

A strong public health framing may inadvertently sideline alternative representations of the issue, particularly those grounded in women’s rights and gender equality. Although rights-based arguments have been present in advocacy efforts, they are often perceived as less culturally acceptable or more politically contentious. As a result, they are used more cautiously, potentially limiting their influence in shaping policy discourse.^39^ Informants expressed mixed views on rights arguments; some noted “*robust discussions on human rights in Malawi*” (KI3 – NGO), while others felt they were underutilised. Kangaude et al. described “pulling back on the human rights argument was perhaps a political miscalculation” (p. 91), especially after the ToP Bill was blocked before Parliament in 2021.^3^

##### Shaping advocacy strategies

The dominance of a public health framing has also shaped advocacy strategies, encouraging the use of evidence-based arguments focused on mortality, morbidity and health system burden.^23^ While this approach has been effective in establishing legitimacy and engaging technical stakeholders, it may constrain the diversity of advocacy approaches, particularly those that rely on moral, legal or rights-based claims.

##### Influencing political acceptability

Positioning abortion law reform as a public health issue has made the topic more politically palatable in a context characterised by strong religious and cultural opposition. By framing reform as a means of reducing preventable deaths rather than as a matter of individual rights, advocates have been able to engage policymakers who might otherwise resist the issue.^26^ However, this strategy may also limit the extent of reform considered politically feasible.

##### Obscuring structural and social dimensions

Finally, a predominantly public health framing tends to obscure the broader structural and social drivers of unsafe abortion, including gender inequality, poverty, stigma and legal constraints. Unsafe abortion disproportionately affects young, rural and poor women who lack accurate information and access to safe abortion care.^42^ This disparity is particularly significant given that approximately 80% of Malawi’s population live in rural areas characterised by high levels of poverty.^43^ Abortion-related stigma remains pervasive throughout Malawian society. Women who have had an abortion are often labelled as murderers, ostracised by their families and communities, excommunicated by religious leaders, and, in some cases, expelled from school.^10^ Stigmatising behaviour also extends to the health care system, where girls and women encounter judgemental treatment and delayed care.^10^ Even parliamentarians who privately support reform have faced slander and political pressure from colleagues. The criminalisation of abortion under the penal code further entrenches this stigma by placing women under a shadow of suspicion and criminality.^44^ Although stigma is gradually declining, as one informant noted, “we *are in a country where ten years ago no one could mention the word abortion. The stigma around it was so huge, now abortion is a centrepiece of discussion on the radio, in newspapers, on Facebook*”, significant barriers persist (KI11 – media). Some improvements in the treatment of post-abortion care patients have been observed following targeted training for healthcare providers.^10^

### How and where is this representation of the “problem” produced, disseminated, and defended? How could it be questioned, disrupted, and replaced?

#### Potential pathways for successful abortion law reform in Malawi

##### Coordinated approach

Formed in 2010, the Coalition on Prevention of Unsafe Abortion (COPUA) has spearheaded abortion law reform advocacy in Malawi. The coalition comprises various civil society organisations, including constituents from women’s, youth and legal organisations, and obstetrician-gynaecologists as well as religious and traditional leaders.^3,22^ As advocacy has become less taboo on the topic, newer and younger organisations have joined, which is a positive development, yet this has led to fragmentation. Coordinated strategies and collaborative work across groups are therefore essential:

“*I feel that COPUA can broaden its membership and learn from the new members. But then the new members should also appreciate that there was a history when no-one wanted to touch abortion, and they are coming in when, the ground is easier, COPUA did that. But sometimes I find that reluctance to work with COPUA*.” (KI13 – INGO)

##### Further evidence

There has been significant evidence demonstrating unsafe abortion incidence and prevalence in Malawi.^7,9,12^ While continued monitoring remains vital, capturing the voices of those affected across diverse demographics, including youth, male partners, married women, those with children, and people of different faiths, is also necessary. Key informants unanimously agreed that personal stories of girls and women effectively generate empathy and understanding. Ongoing data collection would keep local evidence current, highlight the persistent public health impact of restrictive laws despite other maternal health interventions, and counter stereotypes by examining demographic factors (age, marital status, parity, religiosity) associated with unsafe abortion.^4^ Amplifying lived experiences of girls, women, and male partners would further illustrate the realities of restrictive abortion law.

##### Language

Explicit human and women’s rights language has been tested in Malawi, with informants agreeing that rights-based advocacy must continue but be carefully framed to fit the local context – for example, through a non-gendered healthcare lens or emphasis on safe, healthy environments for raising children. Many flagged the Termination of Pregnancy Bill title as incongruous with the Malawian context*: “the moment you talk termination of pregnancy, everybody closes their ears, and they don’t want to listen”* (KI8 – Ministry of Health).

The study findings suggest prioritising disarming language. The current title of the proposed bill immediately closes people off from engaging in constructive dialogue and engagement among lawmakers and community leaders. Reconsidering the bill’s name could be a simple, effective reform strategy. Other African countries have used titles such as “Reproductive Health Law” in Central African Republic^45^ and Niger.^46^ Given Malawi’s strong cultural value on motherhood, a title such as “The Right to Safe Motherhood and Reproductive Health Act” (Nepal), might be more fitting to the local context.^47^

##### Stakeholder engagement

Informants identified the empowerment of local community-based organisations as a priority focus area for future advocacy efforts. This would help to build a critical mass in support for legalising abortion, giving parliamentarians confidence to back reform. Such community-based efforts have proven useful in other contexts, such as Rwanda^48^ and countries in Latin America.^49^ While legal passage rests with MPs, the power to create reform ultimately comes from communities.^50^ Lack of community awareness and engagement was cited as a key reason the ToP Bill has stalled:

“*One of the issues that the Members of Parliament raised when they failed to table the Bill was their fear and that their constituents are not familiar about the laws that are being pursued. And it will be at their cost, they may lose their parliamentary seat if they proceed to table the bill because it’s a controversial bill. So, they requested civil society organisations to say, you need to do more in terms of raising awareness among communities, different constituencies, on the bill. But also, on the need for abortion reform in Malawi.*” (KI14 – NGO)

Where community chiefs have received education on unsafe abortion, they have become powerful allies: “*village traditional leaders, whom we call gatekeepers, they are very supportive now because they have the right information*”. (KI4 – INGO).^3^

Misperceptions persist among community and faith leaders that the ToP Bill would allow for abortion on demand.^50^ A lack of understanding by the general public on the contents of the proposed bill is further impacted by Malawi’s low literacy rates.^43^ Building support requires training of local champions, who are trusted by their communities, be they local chiefs, religious leaders or school headmasters, who can deliver messages in person and more effectively than external health workers who are unknown to the community. In recent years the media have become more vocal in their advocacy efforts for reform, with some journalists partnering with activists to disseminate accurate information.^51^ Long-term strategies include awareness-raising among students in medicine, law and media. Benchmarking with neighbouring countries, who share similar demographics and cultural practices, could help MPs overcome fears. In addition, sensitisation programmes could help to combat misinformation held through mainstream society.

##### Timing

Key informants agreed that timing for reintroducing the ToP Bill to Parliament is critical, though opinions varied on feasibility. As one informant described “*it can be passed, but you need to strategise, you need to understand the politics in Malawi, because you need to know which day a law is easily passed, you need to know who the noisiest members of Parliament are, what's their view on issues of abortion*” (KI3 – NGO).

Another expressed cautious optimism:

“*If there are people that are brave enough, I would have loved if it went to Parliament because they have also recently worked on another Law Commission, we've revised the Public Health Law. Unfortunately, we did not say anything about abortion in this law all we said was that we are supporting what that other law had proposed. So, this law is going to go to Parliament before the end of this year, so I’m hoping probably at that time, somebody may be clever enough to bring in the issue for that other law.*” (KI10 – Ministry of Health)

However, others saw limited opportunity before upcoming elections:

“*Most of the Members of Parliament have one year left. Most of the decisions that go through Parliament go through a voter’s lens, so everyone thinks, if I do this, am I’m going to win the votes or lose votes. So mostly speaking, sensitive issues would not be repeated around when you are close to elections. So, the chances of the bill going to Parliament anytime from now to December 2025 are very slim*.” (KI9 – Health Professional)

Unfortunately, the Global Gag Rule (GGR), which was reinstated by US President Donald Trump in early 2025, may have devastating impacts on advocacy efforts given Malawi’s dependency on US aid.^50^ As seen in 2017, previous reinstatement silenced critical voices, severely disrupted reform efforts, and halted government lobbying.^50^

##### Litigation

The application of strategic litigation has a small and relatively recent history in Malawi.^52^ A 2021 High Court case clarified that preservation of life includes safeguarding the physical and mental health of women faced with an unintended pregnancy.^52,53^ An ongoing High Court case seeks to confirm whether minors who become pregnant due to sexual violence are legally entitled to terminate a pregnancy under existing laws in Malawi.^54^ Pursuing litigation in alternative circumstances where the continuation of pregnancy risks the health and life of women may provide further clarity on the scope of access to legal abortion care in Malawi.^41^ Previous success in related areas, including marriage age and defilement, reinforces the potential of strategic litigation.^23^

#####  Funding

Malawi depends heavily on external donors for reproductive health services, with 70% of funding from abroad.^21^ The United States is the largest bilateral donor to Malawi, making the government reluctant to enact laws that contradict policy stipulations that come with US funding.^50^ One informant suggested reallocation of donor funding streams “*unless the government decided to move USAID to another sector and let UK and Germany take the reproductive health sector. Maybe that’s when things can change”* (KI2 – health professional). Another criticised appeasement towards donors:

“*We cannot have people who think that donors are going to drag the agenda of this country when those that are dying are not donors, they are not from other countries, they are Malawians … we [talk] about donors when Malawians are dying, women are dying.*” (KI1 – NGO)

Internal advocacy requires resources for rural outreach, materials in local languages, training of local champions, and associated travel costs. Funding for advocacy work is especially crucial given that those opposed to reform hold extensive resources and funding. As noted by one informant “*churches have stations dotted throughout the Malawi”* (KI11 – media). The same informant went on to describe the funding in the context of the ToP Bill:

“*The marketing of the [ToP] Bill with adequate resources can be easy, compared to when you don't have those funds, because a church would just write what they call their pastoral letter. Then they will read their pastoral letter throughout Malawi in all their churches.*” (KI11 – media)

##### Path to comprehensive law reform

A 2020 study by the International Women’s Human Rights Clinic (IWHRC) showed an overwhelming majority of Malawian stakeholders (including religious leaders, traditional chiefs, members of government, women’s and youth rights advocates, teachers, academics, medical practitioners, members from NGOs and INGOs) supported abortion on request in the first trimester.^11^ Yet informants and members of the 2013 SLC viewed the full legalisation of abortion (on request) as incompatible with Malawi’s prevailing moral values.^4^ The ToP Bill remains a tangible entry point, but advocates increasingly discuss broader liberalisation.^3^

“*Our view is that if we just push for the grounds that are in the [ToP] Bill, yes it will make some change, but it will not be enough. People will still be accessing unsafe abortions because the majority of their reasons is not defilement, is not incest, is not foetal malformation, it is for reasons that are not in the proposed grounds. More broadly we are looking at comprehensive reform, but we started with pushing for the ToP Bill because it was already recommended by the Malawi Law Commission. So, we thought that was an entry point for pursuing advocacy. But overall, the end goal is comprehensive law reform, where people can access safe and legal abortion on demand, as compared to only restricted grounds.*” (KI14 – NGO)

Despite the proposed ToP Bill being semi-liberal, it would still lead to a meaningful decrease in maternal mortality.^50^ However, if Malawi were to take the next steps towards comprehensive law reform, there is the potential for a 91% decrease in deaths associated with unsafe abortion, which was seen in South Africa following comprehensive reform in 1996.^55^ Despite prolonged resistance, advocates remain hopeful “*it may not be this year, may not be next year, but one day the revolution will come, and it will happen*” (KI1 – NGO).

## Discussion

This study aimed to identify potential pathways for successful abortion law reform in Malawi. Using a multi-method approach involving document analysis and semi-structured interviews with key informants, it examined the policy processes employed by advocates, the influence of those resistant to change, and strategies for advancing more progressive abortion laws. Guided by Bacchi’s “What’s the Problem Represented to be” framework, the findings reveal that advocates have predominantly framed the “problem” of abortion as one of high maternal morbidity and mortality to make reform more politically and culturally acceptable. However, this public health representation operates within deeply entrenched religious and moral presuppositions that continue to dominate public and parliamentary discourse. Despite robust local evidence, sustained advocacy and international pressure, the current restrictive colonial-era law generates significant harms, including high rates of unsafe abortion, pervasive stigma, health system costs and inequities that disproportionately affect rural and poor women. Key informants highlighted several promising pathways forward, including a more coordinated advocacy approach, strategic reframing of language, intensified community engagement through trusted local champions, improved political timing, strategic litigation and sufficient funding to counter well-resourced opposition, while ultimately advocating for comprehensive law reform beyond the proposed Termination of Pregnancy Bill.

Drawing on the findings of the study, a comparative analysis of the drivers for abortion law reform in other Sub-Saharan African countries may help to explain the factors underpinning Malawi’s stagnation. Prior to legal reforms, most countries within SSA held colonial-era laws that criminalised abortion except to save the woman’s life.^56^ This remains the prevailing legal framework in Malawi. Countries which have achieved reform and are also proximate countries to Malawi include South Africa (1996), Ethiopia (2005), Rwanda (2012), Mozambique (2014) and Zambia (1972, amendments in 1994, 2005 and 2017).^48,56–59^ Legal reform in these countries shared several common drivers, including public health concerns arising from high rates of unsafe abortion and maternal mortality; robust domestic advocacy; windows of political opportunity during penal code modernisation or broader legislative transitions; and alignment with human rights commitments.

First, local public health evidence documenting the scale of preventable harm caused by unsafe and illegal abortion has most frequently served as a powerful catalyst for reform efforts. Such evidence has consistently driven calls to reduce maternal mortality through the provision of safe, legal abortion care.^60–63^ In Ethiopia, prior to the 2005 liberalisation, unsafe abortion was a leading cause of maternal mortality, contributing to around 30–32% of all maternal deaths.^61^ This compelling local evidence played a pivotal role in agenda-setting and galvanising support for reform. It provided a strong public health justification that resonated with policymakers and medical professionals, particularly the Ethiopian Society of Obstetricians and Gynecologists (ESOG), who actively used the data to advocate for change during the penal code revision process.^56,61,64^ Although unsafe abortion remains a major public health concern in Malawi, contributing to the country’s high maternal mortality rates, local evidence demonstrating the implications of the country’s restrictive abortion law has failed to enable policy change due to its limited emotional resonance and inability to counter dominant moral and religious narratives.^7,8^ The study findings indicate that strengthening the argument would require supplementing statistical data with nuanced, demographically diverse evidence and compelling personal stories from affected women, girls, and their partners to foster greater empathy and challenge prevailing stereotypes.

A second common driver of change is sustained and effective local advocacy, whereby actors frame unsafe abortion as a matter of public health rather than a moral or ethical issue, thereby gaining support from government leaders and parliament. In Rwanda’s conservative context, reform was driven by youth-led advocacy highlighting the human consequences and public health harms resulting from restrictive abortion laws, with messaging centred on personal narratives of young women imprisoned for seeking or procuring an abortion.^48^ In South Africa, an effective coalition of feminist organisations, health NGOs and activists lobbied intensively during the constitutional and legislative processes.^57,65^ Similar evidence-based framing enabled advocates in Mozambique, Zambia and Ethiopia to navigate religious and conservative opposition.^64,66^ In Malawi, advocates for reform have likewise emphasised unsafe abortion as a public health issue to align with prevailing public opinion. Nevertheless, abortion remains a deeply controversial issue, encountering stiff resistance from opponents, compounded by additional challenges such as a hard-to-reach rural population, low literacy levels, and the substantial influence of well-funded religious organisations within political and community spheres.^12,43^ Strong and well-funded religious opposition to abortion law reform is not unique to Malawi. All comparator countries in this analysis, including Ethiopia, Rwanda, Mozambique, Zambia, and South Africa, encountered similar resistance from religious institutions. What distinguishes Malawi is the relative weakness of countervailing forces, characterised by limited government and civil society collaboration, a lack of high-level political champions, and a more fragmented advocacy movement when confronted with coordinated and financially resourced religious opposition. In contrast, countries that successfully liberalised their abortion laws were able to neutralise or circumvent such resistance through strategic public health framing, effective use of political windows of opportunity, and strong domestic leadership.^48,57,64,67^

Alternative arguments including women’s and reproductive rights, gender discrimination and the substantial public health costs associated with unsafe abortion have also been employed in Malawi. However, these are frequently countered by unsubstantiated claims that prioritise foetal rights or assertions that liberalisation would promote promiscuity.^3,4,50^ Legal reform could significantly reduce health expenditure; Benson et al. (2015) estimated that the annual cost of post-abortion care in Malawi was approximately USD 314,088, a figure that could be reduced by up to 30% with the introduction of safe and legal abortion services in public health facilities.^68^ Notably, Tanzania, which shares a border with Malawi, continues to enforce restrictive colonial-era abortion provisions, inherited from British colonial rule and governed by the Penal Code of 1945.^58^ Like Malawi, Tanzania's lack of reform appears to be attributed to entrenched conservative societal values, the influence of religious institutions and an absence of political will to shift away from colonial legacies.^58^ However, the availability of misoprostol medication through pharmacies and black markets has informally liberalised access to self-managed abortions, enabling women and girls in Tanzania to utilise safer methods in an otherwise highly restrictive legal context.^69^

A critical point of difference between Malawi and other Sub-Saharan African countries that successfully reformed their abortion laws lies in the presence of a window of political opportunity and government receptiveness to change. In countries such as Zambia (following independence in 1964) and South Africa (during the post-apartheid transition), abortion law liberalisation was often embedded within broader penal code revisions or constitutional reforms.^24,57^ This approach helped reduce the political risks associated with introducing standalone controversial legislation.^48,59^ At the time of reform, governments demonstrated openness to modernising laws, aligning with global trends, and advancing women’s status.^24,56,57,70,71^

International pressure also played a supporting role in several comparator countries, where governments responded to regional and international human rights commitments, including the Maputo Protocol. In Malawi, however, despite ongoing pressure from bodies such as the CEDAW Committee and the African Commission on Human and Peoples’ Rights highlighting inconsistencies with its own Constitution and international obligations, such external influences have not translated into domestic reform.^5^ This limited impact stems largely from the perception that international recommendations constitute foreign interference in national sovereignty and cultural values. Religious leaders and conservative politicians have effectively framed such pressure, including recommendations from the CEDAW Committee and the Maputo Protocol, as Western-imposed agendas, thereby undermining their domestic legitimacy.^4,21^

Finally, collaborative relationships between government and civil society were instrumental in successful reforms elsewhere.^56,61^ In Malawi, however, parliamentarians often express private support for reform during discussions with advocates but remain hesitant to endorse change publicly without clear constituent backing. Widespread confusion surrounding the Termination of Pregnancy Bill, coupled with a lack of government ownership and shifting support from religious authorities, has further undermined effective collaboration. In addition, restrictive donor funding policies, particularly those from the United States such as the Global Gag Rule, have further constrained opportunities for meaningful collaboration between the government and civil society actors working to advance abortion law reform.^50^ As a result, the enabling environment of government-civil society partnership and political receptiveness observed in other countries appears notably absent in Malawi.

Key informants in this study proposed a range of practical and contextually relevant strategies to advance abortion law reform in Malawi. These included strengthening coordination between advocacy groups to reduce fragmentation, generating more nuanced demographic and lived-experience evidence to counter stereotypes, reframing the language and title of the Termination of Pregnancy Bill to better resonate with local values (for example, “The Right to Safe Motherhood and Reproductive Health Act”), intensifying community sensitisation through trusted local champions such as chiefs and religious leaders, strategic political timing, targeted litigation to clarify and expand existing legal provisions and securing sufficient funding to match the well-resourced opposition. These suggestions appear largely on the right track when viewed through the comparative experiences of other Sub-Saharan African countries. Successful reforms in Ethiopia, Rwanda, Mozambique, Zambia, and South Africa were consistently driven by strong public health evidence, effective domestic advocacy coalitions, harm-reduction framing, community engagement and strategic use of political windows. However, the comparative analysis also reveals two critical gaps in the Malawian context: genuine government ownership and receptiveness to reform, and the presence of a conducive political opportunity. While the strategies identified by key informants are necessary and promising, they are unlikely to succeed in isolation. Sustainable progress towards comprehensive abortion law reform in Malawi will ultimately depend on improved collaboration between government and civil society, coupled with the emergence of a favourable political moment that allows evidence and advocacy to overcome deeply entrenched religious and political resistance.

Moreover, it is essential to recognise that legal frameworks and policies are just one component of a complex interplay of factors that determine access to safe abortion care. Consequently, should the ToP Bill be enacted, advocates must prioritise effective implementation. This would entail widespread awareness-raising among the public and health care providers, alongside the dissemination of clear clinical guidelines to ensure consistent application of the law. Ongoing advocacy efforts should also concentrate on sustained destigmatisation initiatives, with particular emphasis on addressing negative attitudes and obstructionist behaviours among healthcare professionals, factors that frequently impede service delivery even in permissive legal environments. Furthermore, to build a robust case for more comprehensive law reform in the future, rigorous monitoring and evaluation mechanisms will be indispensable. These should pay special attention to those groups of girls and women who are likely to remain excluded from safe and legal abortion care under the provisions of the proposed legislation, a cohort that is expected to remain substantial.

### Study limitations

As with any retrospective research, this study has limitations, including recall bias, researcher bias, and social desirability bias. Recall bias may have occurred during the interview process. Our discussions were based on experiences, opinions and observations of those who generally had a long and rich history of involvement in the abortion law reform process in Malawi. We tried to mitigate recall bias by employing a multi-method approach, using a comprehensive document analysis of both peer-reviewed and grey literature to triangulate the findings. Three researchers (CH, JD & DH) were all involved in the continuous development of the data collection processes and analysis as a means of mitigating any potential researcher bias and ensuring data collection methods remained relevant and effective. In addition, social desirability bias may have influenced the accuracy and validity of interview data. We tried to mitigate social desirability bias by interviewing a wide variety of informants, from different professional sectors, and also by ensuring participants' anonymity and confidentiality throughout their involvement in the study. It should also be noted that while the WPR framework was originally designed for documentary policy analysis, it can be used for interview analysis, but examples in the literature of its application are limited. Throughout the study, every effort was made to address all concepts covered by the six questions of the WPR framework. However, the use of deductive followed by inductive qualitative analysis of both document and interview data may have resulted in incomplete coverage of some concepts within the WPR questions. Furthermore, generalisability of the findings may be limited given Malawi’s context and unique abortion law reform history.

## Conclusion

This study examined the policy processes adopted by advocates seeking abortion law reform in Malawi, while also exploring potential pathways for future progress. Employing a multi-method case study design, the research drew on qualitative data gathered through semi-structured interviews and document analysis, adhering to SRQR reporting guidelines for rigorous analysis, triangulation, and transparent presentation of findings. Malawi has a long history of committed grassroots advocacy in support of safe abortion access. Yet opposition, predominantly grounded in religious doctrine, retains substantial influence across Malawian society, extending from rural communities to the highest levels of political leadership. Well-resourced religious institutions, supported by established networks and effective community outreach, continue to disseminate misinformation about abortion, reinforcing resistance to change. Despite these formidable barriers, advocates persist in their efforts to end preventable maternal suffering and uphold the rights and well-being of women and girls in Malawi. The findings of this study, combined with a comparative analysis of reform experiences in neighbouring and regional Sub-Saharan African countries (Ethiopia, Rwanda, South Africa, Mozambique, and Zambia), highlight the primary obstacles to comprehensive legislative reform in Malawi. These obstacles include entrenched religious opposition and cultural stigma; restrictive external donor funding largely originating from the United States, which shapes government and civil society priorities and spending; and fragile political support for abortion law change.^21^ By documenting the perspectives and experiences of diverse actors operating within this constrained environment, the study contributes to the limited understanding of abortion policy processes in restrictive settings. These insights offer practical guidance for advocates in Malawi and provide transferable lessons for those working towards safer abortion laws in similarly challenging contexts worldwide.

## Supplementary Material

S2. Document Data Extraction Sheet

S1. Interview Guide

SRQR guideline checklist

## Provenance statement

This article was not commissioned and went through external peer review.
