# Supplementary material for: Rethinking abortion law reform in Malawi: a multi-method study
Source: Sex Reprod Health Matters. 2026 Jul 27;33(1):2702233. doi: 10.1080/26410397.2026.2702233 (PMC13501753; doi:10.1080/26410397.2026.2702233)
Supplement: Supplementary file 2 [file ZRHM_A_2702233_SM7211.docx]

**S1. Interview Guide**

Pre-Interview checklist:

- The participant has read and understands the information form.
- The participant has read, understands and signed their consent form
- The participants has been asked if they have any questions or concerns

| WPR framework questions | | Guiding questions |
| --- | --- | --- |
| 1 | What’s the problem represented to be in a specific policy or policies? | In advocating for abortion law reform in Malawi, the problem was represented as a public health issue, specifically that of high maternal mortality. Thus, abortion law reform was viewed as a means to reduce maternal mortality rates. How else can the argument be framed?   a. *On the grounds of women’s reproductive and bodily autonomy* b. *Reproductive health rights* c. *Global/regional reproductive health agreements* |
| 2 | What deep-seated presuppositions or assumptions (conceptual logics) underlie this representation of the ‘problem’ (problem representation)? | Why did those advocating for abortion law reform adopt a public health argument as a need for abortion law reform? |
| 3 | How has this representation of the ‘problem’ come about? | How did the public health argument for reform come about?   a. *Stemmed from transnational influences and domestic advocacy; global norms; evidence of the magnitude of maternal mortality in Malawi; evidence of the health care costs associated with unsafe abortion; public health impacts of unsafe abortion.* |
| 4 | What is left unproblematic in this problem representation? Where are the silences? Can the ‘problem’ be conceptualised differently? | How could the _________ be used to reframe the argument for abortion law reform?   a. *Gender discrimination* b. *Reproductive health rights/justice* c. *Sexual and reproductive autonomy* |
| 5 | What effects (discursive, subjectification, lived) are produced by this representation of the ‘problem’? | What are the implications of the current abortion law on women and girls in Malawi?   a. *High incidence of unsafe abortion* b. *Stigma associated with abortion* c. *Social and economic effects on women and girls* d. *Equity issues (safe abortion services are limited, centralised to urban areas, often provided by private clinics and NGOs)* |
| 6 | How and where has this representation of the ‘problem’ been produced, disseminated and defended? How has it been and/or how can it be disrupted and replaced? | How can __________ (*identified implications*) be used to frame the need for reform today? Where and how have they been used in replacement of the public health argument? |

Post-Interview checklist:

- The participant has been reminded that they will receive a copy of the revised transcript and are free to redact or remove anything
- The participant has been asked for references to supporting documents
- The participant has been asked to provide contact information for potential participants
- The participant has been thanked for their time and for generously sharing their experiences and insights to help with the study
