# Supplementary material for: Rethinking abortion law reform in Malawi: a multi-method study
Source: Sex Reprod Health Matters. 2026 Jul 27;33(1):2702233. doi: 10.1080/26410397.2026.2702233 (PMC13501753; doi:10.1080/26410397.2026.2702233)
Supplement: Supplementary file 3 [file ZRHM_A_2702233_SM7695.docx]

| Full reference | Document title | Document type | Summary of key document analysis findings |
| --- | --- | --- | --- |
| Benson, J., Gebreselassie, H., Mañibo, M. A., Raisanen, K., Johnston, H. B., Mhango, C., & Levandowski, B. A. (2015). Costs of postabortion care in public sector health facilities in Malawi: A cross-sectional survey. BMC Health Services Research, 15(1), 562-562. doi:10.1186/s12913-015-1216-2 | Costs of postabortion care in public sector health facilities in Malawi: a cross-sectional survey. | Peer-reviewed Journal Article | - Estimates health system costs of treating unsafe abortion complications and compares findings with costs for providing proposed safe abortion care in Malawi - Including in-depth surveys at 15 purposively selected public health facilities providing postabortion care. - Postabortion care treatment in public facilities cost an estimated $314,000 annually - Transition to safe, legal abortion care would yield an estimated cost reduction of 20-30%. - The method and severity of complications had a large impact on overall costs - A liberalisation and implementation of safe abortion law could markedly decrease cost to the Malawian health care system |
| Center for Reproductive Rights. (2021). High Court of Malawi Clarifies Law on Abortion [Press release]. Retrieved from https://reproductiverights.org/high-court-of-malawi-clarifies-law-on-abortion/ | High Court of Malawi Clarifies Law on Abortion | Press Release | - Press release on ruling made in the Malawi High Court clarifying section 243 of the Penal Code which makes an exception for abortion in situations where the life and health of a woman or girl is in danger. - The Court recognised that the preservation of life also entails safeguarding the mental and physical health of the woman or girl - Advocates welcomed the ruling as it provided clarity for health care providers, but they also used the media opportunity to explain that regardless of the clarification the current law still violates human rights |
| Chiputula, E. (2023, 23 October 2023). Rights organizations support 14-year-old girl’s court action on pregnancy termination. The Atlas. https://atlasmalawi.com/rights-organizations-support-14-year-old-girls-court-action-on-pregnancy-termination/ | Rights organisations support 14-year-old girls court action on pregnancy termination | News Article | - Media article on current High Court case concerning 14-year-old girls right to an abortion resulting from sexual violence in Malawi - Those seeking justice aim to for legal clarification according to sections within the Gender Equality Act - The case holds immense public importance, concerning the ability of pregnant minors and survivors of sexual violence to access safe and legal abortion care as per the current law - Advocates believe that when a girl or woman becomes pregnant due to sexual violence, her life is in danger, and hence would fall within the current legal exception to access abortion services if they so choose - Advocates are hopeful that the court determination would provide much needed clarity on the law as well as the legal obligations required by healthcare professionals when caring for those who have experience sexual assault resulting in pregnancy |
| Daire, J., Kloster, M. O., & Storeng, K. T. (2018). Political Priority for Abortion Law Reform in Malawi: Transnational and National Influences. Health and Human Rights, 20(1), 225. https://www.ncbi.nlm.nih.gov/pmc/articles/PMC6039725/ | Political Priority for Abortion Law Reform in Malawi: Transnational and National Influences | Peer-reviewed Journal Article | - Prospective policy analysis, identifying factors that helped generate political will to address unsafe abortion in Malawi - Discusses transnational influence and domestic advocacy, making unsafe abortion a political issue in Malawi, and bolstering the possibility of abortion law reform - Explores promotion of global norms and the provision of financial and technical resources in support of advocacy efforts - Advocates frame action on unsafe abortion as part of a broader imperative to address Malawi’s high maternal mortality - Considers backlash from those opposed to abortion and the potential for the ToP Bill to antagonise the United States as Malawi’s primary external health aid funder |
| Jackson, E., Johnson, B. R., Gebreselassie, H., Kangaude, G. D., & Mhango, C. (2011). A strategic assessment of unsafe abortion in Malawi. Reproductive Health Matters, 19(37), 133-143. doi:10.1016/S0968-8080(11)37563-5 | A strategic assessment of unsafe abortion in Malawi | Peer-reviewed Journal Article | - Broad scale qualitative study, including 485 interviews in-depth interviews with Malawians on sexual and reproductive health, maternal mortality and unsafe abortion - Provides additional review of human rights-based in the context of Malawi’s laws, policies and international agreements related to sexual and reproductive health - Significant barriers which perpetuate unsafe abortion included Malawi’s restrictive abortion law, inaccessibility of safe abortion services, particularly for poor and young women, a lack of adequate family planning and post-abortion care services - Urgent action areas identified included abortion law reform; sexuality education and family planning; adolescent sexual and reproductive health services; and post-abortion care services |
| Kalilani-Phiri, L., Gebreselassie, H., Levandowski, B. A., Kuchingale, E., Kachale, F., & Kangaude, G. (2015). The severity of abortion complications in Malawi. International Journal of Gynecology and Obstetrics, 128(2), 160-164. https://doi.org/10.1016/j.ijgo.2014.08.022 | The severity of abortion complications in Malawi | Peer-reviewed Journal Article | - Quantitative study into the severity of abortion complications in Malawi and to determine associated risk factors - Researchers conducted a cross-sectional survey at 166 facilities providing postabortion care services in Malawi - 80.9% of women who presented for postabortion care in 2009 were married and 64.8% were from rural areas - The most common complications included sepsis, retained products of conception and fever - Women with severe or moderate complications were significantly more likely to be from rural areas than from |
| Kamanga, P. P. (2024, 11 March 2024). Young girls bear the brunt of unsafe abortion. All Africa. https://allafrica.com/stories/202403110028.html | Young girls bear the brunt of unsafe abortions | News Article | - Media article discussing that most of the post-abortion care following unsafe abortion is providing to young girls, specifically those under or slightly over the age of 20 years - Highlights the vulnerability of adolescent girls and young women to the risks associated with unsafe abortion - Discusses proposed ToP Bill and engagement of sensitisation of stakeholders |
| Kangaude, G. D., & Mhango, C. (2018). The duty to make abortion law transparent: A Malawi case study. International Journal of Gynecology and Obstetrics, 143(3), 409-413. https://doi.org/10.1002/ijgo.12630 | The duty to make abortion law transparent: A Malawi case study. | Peer-reviewed Journal Article | - Authors argue that an immediate step which the government of Malawi must take is clarify the current abortion law, regardless of potential for proposed legislation to materialise in the indeterminate future - The article explores Malawi’s current law through the lens of a case study of a 13-year-old who became pregnant because of a sexual crime - The article draws upon the obligations under national and international law, to ensure that access to legal abortion is realised in accordance with the law, in addition to providing clear and transparent guidance for healthcare providers currenting operating in Malawi |
| Kangaude, G. D., Sibande, C. G., Deller Ross, S., & Xiao Liu, M. (2023). Abortion law reform in Malawi: a case study in stakeholder engagement, public education, and human rights advocacy. Research Handbook on International Abortion Law, 82-102. doi:doi.org/10.4337/9781839108150.00013 | Abortion law reform in Malawi: a case study in stakeholder engagement, public education, and human rights advocacy | Book (Chapter) | - Review of the country’s efforts to reform the 160-year-old abortion law in Malawi - Focus on the strategies employed by the Coalition for the Prevention of Unsafe Abortion (COPUA) - Considers how advocates for reform operated against a backdrop of entrenched cultural and religious attitudes towards abortion - Discussion on the proposed ToP Bill and how attempts to present the Bill to parliament were blocked by those opposed - Discussion on how the current law violates women’s human rights and legal strategies, including litigation |
| Levandowski, B. A., Kalilani-Phiri, L., Kachale, F., Awah, P., Kangaude, G., & Mhango, C. (2012). Investigating social consequences of unwanted pregnancy and unsafe abortion in Malawi: The role of stigma. International journal of gynaecology and obstetrics, 118(2), S167-S171. doi:10.1016/S0020-7292(12)60017-4 | Investigating social consequences of unwanted pregnancy and unsafe abortion in Malawi: The role of stigma | Peer-reviewed Journal Article | - Investigates the social consequences of unwanted pregnancy and unsafe abortion in Malawi in the context of stigma - In-depth interviews were conducted with 486 Malawian stakeholders belonging to different political and social structures - Interviewees identified the impact of unwanted pregnancy and unsafe abortion to be the greatest on young women - Premarital and extramarital pregnancies were highly stigmatised and access and use of contraception in Malawi is challenging - The researchers concluded that community-level discussions focusing on the reduction of stigma would be beneficial |
| Ligomeka, B. (2024). The power of religious voice in abortion law reform advocacy: interfaith approaches to abortion law reform in Malawi. In F. Bloomer & K. Turtle (Eds.), Reimagining Faith and Abortion A Global Perspective (pp. 81-96). Bristol University Press. https://doi.org/10.56687/9781447370178-008 | The power of religious voice in abortion law reform advocacy: interfaith approached to abortion law reform in Malawi | Book Chapter | - Acknowledges abortion as a polarising moral issue that is heavily stigmatised in Malawi - Considers the influence and power of religion despite being a secular state - Malawi has maintained UK Offences Against the Person Act of 1861, brought to Malawi during colonialism, yet Britian and Northern Ireland have heavily reformed abortion law since - Considers different religious perspectives on abortion, unpacks meaning behind religious scriptures and standard religious beliefs - Considers religious leaders as advocates, noting trained and empowered religious leaders can be powerful allies in abortion law reform, working to neutralise propaganda advanced by others in the name of religion - Malawi is not a theocracy, but a secular state that, on paper, embraces religious freedoms and cultural diversity, there is the need to liberalise abortion law as part of reducing maternal mortality |
| Liu, M. X., & Sudhakar, N. (2021). Between a Rock and a Hard Place: The Global Gag Rule's Impacts on Women's Reproductive Health and Abortion Law Reform in Malawi. University of Pennsylvania Journal of International Law, 43, 469. Retrieved from https://scholarship.law.upenn.edu/cgi/viewcontent.cgi?article=2043&context=jil | Between a Rock and a Hard Place: The Global Gag Rule's Impacts on Women's Reproductive Health and Abortion Law Reform in Malawi | Report | - Examines the harmful impacts of the Global Gag Rule in the Malawi context - The rule puts in place conditions for those agreeing to receiving US Global health foreign aid. Recipients will not perform or support abortion as a method of family planning - The policy also prohibits NGOs from conducting safe abortion advocacy, thereby stifling education and awareness activities related to abortion law reform - The report, published in the US, argues that legislation must be enacted to stop the policy given its global impact on the rights and lives of girls and women in Malawi and around the world |
| Malawi Law Commission. (2016). Report of the Law Commission on the review of the law on abortion in Malawi. https://www.lawcom.gov.mw/sites/default/files/Law%20Commission%20Report%20on%20the%20Review%20of%20the%20Law%20on%20Abortion.pdf | Report of the Law Commission on the review of the law on abortion in Malawi | Government Report | - Official Law Commission Report on the review of the law on abortion - Lists members and associations of those involved in the Commission - Discusses background information on abortion, history of abortion law and additional policies which relate to abortion in Malawi - Considers the magnitude of the unsafe abortion in Malawi and globally, foetal rights versus women’s reproductive rights, international and regional legal frameworks on abortion and provides a justification for reform. - Clarifies that the SLC agreed that the law on abortion should be liberalised on specific grounds, including life, physical and mental health, malformation of the foetus, and rape, incest or defilement. Provides specific detail on each of these grounds. - Also specifies the grounds upon which abortion is not legally allowed - Provides information on the implementation of the proposed law and how this would transfer into safe abortion care services in Malawi, addresses supplementary information including conscientious objection, consent etc. - Provides draft Termination Pregnancy Bill and guidelines for service delivery |
| Masina, L. (2021, 19 June 2021). Malawi Parliament Withdraws Abortion Rights Bill after Objections. Voice of America. https://www.voanews.com/a/africa_malawi-parliament-withdraws-abortion-rights-bill-after-objections/6207221.html | Malawi Parliament Withdraws Abortion Rights Bill after Objections | Media Article | - Published following the second attempt to reintroduce the ToP Bill in 2021 - The bill was withdrawn from Malawi’s parliament, due to stiff resistance from lawmakers and citizens, but primarily anti-abortion voice - MP Mathews Ngwale who was tasked with presenting the bill indicates a lack of confidence. Noting that work need to be done within communities to instil confident in lawmakers that the public are in support of change to the abortion law - Provides evidence-based statistics of unsafe abortion rates - ‘One of the latest is the death of a 14-year-old girl, after she took a herbal concoction in an attempt to terminate a five-month pregnancy’ - Clarification on the specific legal grounds the ToP Bill would provide - Amos Nyaka, deputy chairperson for COPUA, discusses the use of litigation given a lack of confidence in parliamentarians being moved into action to change the law |
| Mzungu, W. (2024, 21 March 2024). Journalists asked to question government why Termination of Pregnancy Bill is not being tabled in Parliament. Nyasa Times. Retrieved from https://www.nyasatimes.com/media-asked-to-question-govt-why-termination-of-pregnancy-bill-is-not-being-tabled-in-parliament/ | Journalists asked to question government why Termination of Pregnancy Bill is not being tabled in Parliament | Media Article | - A Consortium of various civil society organisations appeal to the media to question why the government is reluctant to table the Termination of Pregnancy Bill in Parliament - The consortium includes members from sexual and reproductive rights NGOs, human rights NGO, media and COPUA - Advocates vow to continue pushing for the Bill to be tabled given the impacts of unsafe abortion on girls and women in Malawi - Demonstrates advocates use of media to disseminate correct information on safe abortion and reasons why the ToP Bill needs to be pursued |
| Ngwena, C. G. (2016). Taking Women's Rights Seriously: Using Human Rights to Require State Implementation of Domestic Abortion Laws in African Countries with Reference to Uganda. Journal of African Law, 60(1), 110-140. https://doi.org/10.1017/S002185531500025X | Taking Women's Rights Seriously: Using Human Rights to Require State Implementation of Domestic Abortion Laws in African Countries with Reference to Uganda | Peer-reviewed Journal Article | - Using Uganda as a case study, the article calls out the failure of governments to provide women legal and accessible abortion services, which are in clear violation of human rights - The author argues that states take positive steps to implement domestic abortion law as a deliberate advocacy strategy - The article focuses on the implementation of existing domestic laws not the broadening of legal grounds and draws upon international human rights frameworks |
| Pensulo, C. (2021, 25 February 2021). Malawi MPs debate bill to liberalise abortion laws as churches oppose. The Guardian. https://www.theguardian.com/global-development/2021/feb/25/malawi-mps-debate-bill-to-liberalise-abortion-laws-as-churches-oppose#:~:text=Pro%2Dchoice%20campaigners%20believe%20almost,attempt%20to%20change%20the%20law. | Malawi MPs debate bill to liberalise abortion laws as churches oppose | Media Article | - Media update regarding the second attempt to present the ToP Bill in 2021 - Clarifies current legality of abortion in Malawi and the proposed grounds to be included in the ToP Bill - Strong opposition from religious authorities who believe the bill does not reflect the cultural values of Malawi - Provides evidence-based statistics of unsafe abortion rates and some of the dangerous methods used to induce abortion in Malawi - Advocates point out that the debate about abortion is not a religious argument, it is about saving women’s lives |
| Polis, C. B., Mhango, C., Philbin, J., Chimwaza, W., Chipeta, E., & Msusa, A. (2017). Incidence of induced abortion in Malawi, 2015. PLoS ONE, 12(4), e0173639-e0173639. doi:10.1371/journal.pone.0173639 | Incidence of induced abortion in Malawi | Peer-reviewed Journal Article | - Study involved representative surveys of health facilities to estimate the number of cases of postabortion care, in addition to a survey of informants to estimate the probability of needing and obtaining postabortion care - Results provided estimates of induced abortion and unintended pregnancy in Malawi - The study showed that approximately 141,044 induced abortions occurred in Malawi in 2015, 53% of pregnancies were unintended and 30% of unintended pregnancies ended in abortion - Results demonstrated a real need to provide for safe and legal abortion, to prevent the impacts of unsafe abortions deaths in Malawi |
| Sexuality Policy Watch. (2015, 28 July 2015). Unpacking Malawi Special Law Commission final review’ findings and recommendations on abortion law https://sxpolitics.org/unpacking-malawi-special-law-commission-final-review-findings-and-recommendations-on-abortion-law/13151 | Unpacking Malawi Special Law Commission final review’ findings and recommendations on abortion law | Press Release | - The current position of the law is that abortion is illegal in Malawi except where it is performed to save the life of the pregnant women through a surgical operation - The Special Law Commission resolved and agreed that abortion law in Malawi should be liberalised (conditional relaxation of the restrictions) as opposed to decriminalisation to cater for certain justifiable instances where termination of a pregnancy should permissible - Considers current punishments stated in the penal code in relation to abortion - The government of Malawi, with funding from Ipas instituted a Special Law Commission to review the abortion law, comprising of various representatives - Clarifies proposed grounds and supplementary requires recommended by the commission |
| Southern Africa Litigation Centre. (2021). Malawi’s obligation to enact the Termination of Pregnancy Bill. https://www.southernafricalitigationcentre.org/2021/09/27/malawis-obligation-to-enact-the-termination-of-pregnancy-bill/ | Malawi’s obligation to enact the Termination of Pregnancy Bill | Press release | - Media article concerning blocking of the ToP Bill by ultra-conservative members of parliament in 2021 - Highlights a lack of discussions addressing the impact of the continuation of the current law on the rights and livelihoods of women and girls in Malawi - Provides clarification on the current law on abortion and its criminalisation within the Malawian Penal Code, including the ambiguous exception regarding to preserve life - Provides shortfalls of the current law and implications on girls and women - Identifies Malawi’s failure to adhere to human rights and other regional instruments, including the Maputo Protocol, as well as national policy - Provides clarity on the specific legal grounds the ToP Bill |
| WLSA Malawi, Kangaude, G., & IWHRC. (2020). Through Her Eyes: The Harms of Abortion Criminalisation and the Need for Reform. G. Law. www.law.georgetown.edu/wp-content/uploads/ 2020/10/Through-Her-Eyes-The-Harms-of-Abortion-Criminalisation-and-the-Need-for-Reform.pdf | Through Her Eyes: The Harms of Abortion Criminalisation and the Need for Reform | Report | - Report summarising extensive research on the rights guaranteed by Malawi’s constitution, as well as the rights enshrined in various international and regional treaties to which Malawi is a State Party - Intensive fact-finding investigation conducted in Malawi, involving 70 interviews with various stakeholders - Discusses the current law, its implementation and history - Considers the harmful consequences of abortion criminalisation - Discusses the underlying reasons for unintended pregnancy, rights violations under international law and the constitution - Provides recommended changes to the ToP Bill |
